# Single-Cell Sequencing-Based Validation of T Cell-Associated Diagnostic Model Genes and Drug Response in Crohn’s Disease

**DOI:** 10.3390/ijms24076054

**Published:** 2023-03-23

**Authors:** Zhujiang Dai, Jie Zhang, Weimin Xu, Peng Du, Zhongchuan Wang, Yun Liu

**Affiliations:** 1 Department of Colorectal Surgery, Xinhua Hospital, Shanghai Jiaotong University School of Medicine, Shanghai 200092, China; 2 Shanghai Colorectal Cancer Research Center, Shanghai 200092, China; 3 Department of Gastroenterology, Xinhua Hospital, Shanghai Jiaotong University School of Medicine, Shanghai 200092, China

**Keywords:** Crohn’s disease, immune landscape, T cell, single-cell RNA sequencing, molecular docking

## Abstract

Crohn’s disease is a highly heterogeneous autoimmune disease with a unique inflammatory phenotype of T cells at the lesion site. We aim to further explore the diagnosis of Crohn’s disease and drug prediction of T cell marker gene expression. We obtained single-cell expression profile data from 22 CDs or normal samples and performed cell annotation and cellular communication analysis. Through the intersection of T cell marker genes, differential genes, and WGCNA results, we identified T cell-specific key genes and their immune landscapes and potential pathogenesis, and validated them across multiple datasets and patient tissue samples. We also explored the differentiation characteristics of genes by pseudo-temporal analysis and assessed their diagnostic performance and drug sensitivity by molecular docking. Finally, we extended this study to the prognosis of IBD-associated colon cancer. TNF-centered 5-gene diagnostic model not only has excellent diagnostic efficacy, but is also closely associated with KRAS, P53, and IL6/JAK/STAT3 pathways and physiological processes, such as EMT, coagulation, and apoptosis. In addition, this diagnostic model may have potential synergistic immunotherapeutic effects, with positive correlations with immune checkpoints such as CTLA4, CD86, PDCD1LG2, and CD40. Molecular docking demonstrated that BIRC3 and ANXA1 have strong binding properties to Azathioprine and Glucoocorticoid. Furthermore, the 5-gene model may suggest antagonism to IFX and prognosis for colon cancer associated with inflammatory bowel disease. Single-cell sequencing targeting T cell-related features in patients with Crohn’s disease may aid in new diagnostic decisions, as well as the initial exploration of high-potential therapies.

## 1. Introduction

The global prevalence of inflammatory bowel disease (IBD), a persistent inflammatory disease of the intestine, is increasing annually. In particular, patients in newly industrialized cities or regions are experiencing an increased unemployment rate (20–25%) due to the increased burden of this disease [1,2]. The specific pathogenesis of this autoimmune disease remains elusive and has been attributed to a combination of genetic susceptibility, immune microenvironment, external factors, and intestinal microbiota [3,4,5]. Notably, although a new treatment era for IBD has opened up with infliximab (IFX), adalimumab (ADA), and certolizumab, etc., 20% of the population is still unresponsive to anti-TNF therapy. More importantly, further optimization of personalized induction and maintenance (long-term and even lifelong) remains a major challenge. IBD includes two distinct phenotypic subtypes, Crohn’s disease (CD) and ulcerative colitis (UC). Celiac disease is a partially overlapping symptom of CD and ulcerative colitis, and is thought to be mediated by a dysregulated T-cell response [6]. Interestingly, differential diagnosis of subtypes can depend on a limited but distinct subset of T cells or markers [7]. Single-cell RNA sequencing (scRNA-seq) of T cells reveals heterogeneity in gene expression between homologous or individual cells [8]. T-cell gene profiling by high-throughput sequencing is increasingly being applied to identify T-cell and antigenic triggers in diseases and to develop new therapeutic strategies [9]. It is well known that one of the pro-inflammatory hypotheses of IBD, including CD, is a dysregulated or disturbed T-cell responsiveness to intestinal antigens. Moreover, with the iteration of single-cell sequencing technologies, we are more eager to explore new T cell-related diagnostic or therapeutic targets to enrich existing potential candidates for immunomodulators and biologics. Our starting point is to identify and annotate the characterization and enrichment of T cell-associated genes in CD by integrating scRNA-seq data, simulating their stage-specific changes in T-cell development, and constructing and validating a multigene diagnostic model. Finally, we performed molecular docking of key genes to assess the feasibility of drug therapy and extended it to IBD associated colon cancer to assess its prognosis.

## 2. Results

### 2.1. Single-Cell Clustering and Cell Annotation

The overall flow of this study is presented in Graphical Abstract. After normalizing the filtered single-cell data by log-normalization, we used the FindVariableFeatures function to search for highly variable genes and eliminate the batch effect between samples through the FindIntegrationAnchors function (Figure S3 and Table S2). After data integration, we performed PCA dimensionality reduction and found that the PC dimension flattened after 40 (Figure S4). Therefore, we chose dim = 40 and clustered the cells (resolution = 0.4) to obtain 19 subsets (Figure S5).

We obtained markers of gut and immune cells from previous studies [10,11,12,13,14], including epithelial cells (EPCAM, KRT8, KRT18, and FABP1), fibroblasts (COL1A1, COL1A2, and COL3A1), T cells (CD3D, CD3E, CD3G, and CD2), B cells (CD37, MS4A1, VPREB3, and CD79A), plasma cells (SSR4, IGLL5, and IGLL1), mast cells (TPSAB1 and TPSB2), NK cells (KLRF1 and NKG7), myeloid cells (LYZ, C1QA, and CD163), and endothelial cells (PECAM1, VWF, CLDN5, and CDH5).

**Figure 1:**
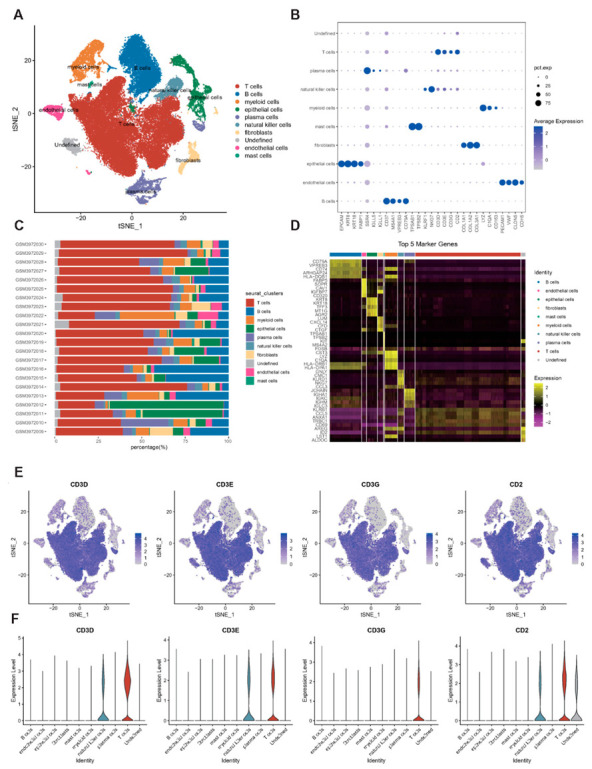
Cell annotation based on single cell data. (**A**): T-SNE plot of cell annotation after single-cell downscaling clustering. (**B**): Expression bubble plot of cell maker genes. (**C**): Percentage of different cells in each sample. (**D**): Heat map display of different maker genes in different cells. (**E**): Display of maker genes of T cells in T-SNE. (**F**): Violin plot of maker genes of T cells in different cells.

We reclassified the marker gene into ten cells based on its expression in the cluster, one of which was undefined (Figure 1A). A bubble plot of marker gene expression in cells is displayed in Figure 1B. In addition, we found large differences in the proportion of cells in different samples (Figure 1C), indicating that there is a greater cell heterogeneity in different patients with CD. We used the FindAllMarkers function to identify the unique maker gene in each cell and filtered it (FC > 1.2 and FDR < 0.05, Figure 1D). In order to prove the accuracy of clustering more intuitively, we plotted T-SNE distribution and expression of each marker gene in the cells. Here, we depict the distribution of only four marker genes (CD3D, CD3E, CD3G, and CD2) of T cells (Figure 1E,F, Figures S6 and S7).

### 2.2. Cell Communication Analysis Related to T Cells in scRNA-Seq

**Figure 2:**
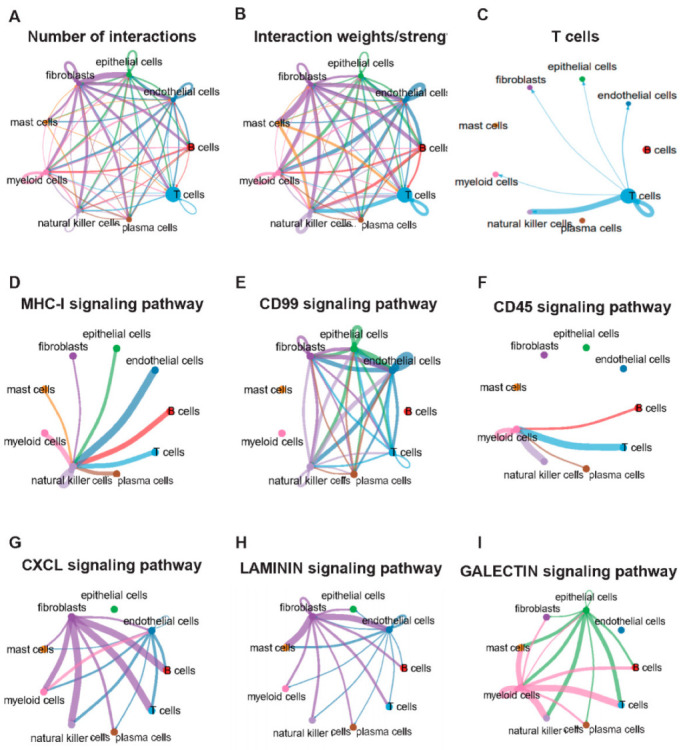
Cellular communication is based on a single-cell level. (**A**): Direction and number of cell communication. (**B**): Direction and intensity of communication between cells. (**C**): Direction and intensity of active communication between T cells and others. (**D**–**I**): Communication network diagram of MHC-I, CD99, CD45, CXCL, LAMININ, and GALECTIN signaling pathway between cells.

Nine known cell types (epithelial cells, fibroblasts, T cells, B cells, plasma cells, mast cells, NK cells, myeloid cells, and endothelial cells) were identified in CD samples. However, sample or cell-to-cell heterogeneity may alter their interactions. To explore the cellular interaction characteristics of CD, we performed a cellular communication analysis based on single-cell data and cell classification combined with CellChat’s built-in CellChatDB.human as a reference. The results revealed that there was complex communication between different cells (Figure 2A,B). T cells mainly communicate with NK cells, epithelial cells, fibroblasts, myeloid cells, and endothelial cells; however, the intensity of communication with NK cells is greater (Figure 2C). Furthermore, we analyzed the communication between 32 pathways in cells. There is large heterogeneity in the amount and intensity of signal exchange between cells in different pathways. We enumerated six signaling pathways in which T cells participate, including the MHC-I, CD99, CD45, CXCL, LAMININ, and GALECTIN signaling networks (Figure 2D–I). In the CD99 signaling pathway, T cells were closely related to endothelial cells and had a weak relationship with NK cells (Figure 2D).

### 2.3. T-Cell Enrichment Analysis in scRNA-Seq

**Figure 3:**
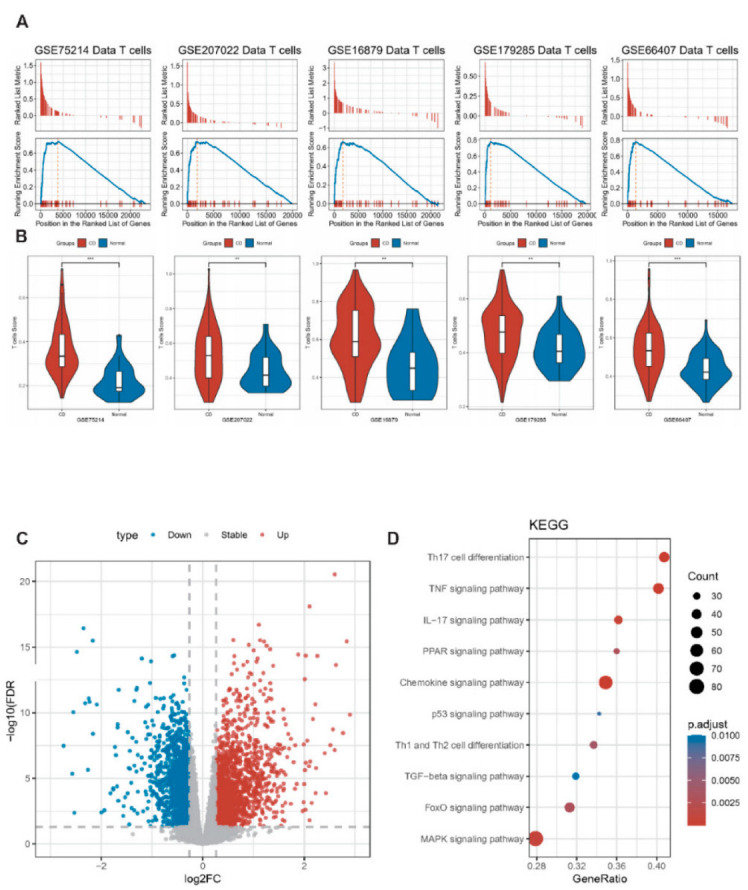
Multi-dataset T-cell enrichment score and identification of differential genes. (**A**): T-cell enrichment analysis results of GSEA analysis for five datasets. (**B**): T-cell differences in CD and normal for ssGSEA analysis for five datasets. ** represents *p* < 0.01, *** represents *p* < 0.001 (**C**): Volcano plot of differential genes for CD and normal subgroups in dataset GSE75214. (**D**): Partial KEGG enrichment results of differential genes for CD and normal subgroups in dataset GSE75214.

Using single-cell data, we identified cell types and analyzed their marker genes, of which T cells had 55 marker genes (Table S2). To verify the distribution of the marker genes in CD, we assessed T cell enrichment in CD and normal samples based on GSEA function. The results demonstrated that T cells were significantly enriched in CD samples from the above five independent datasets (Figure 3A). Moreover, we calculated T cell score for each sample separately by ssGSEA method and compared the difference in T cell scores between CD and normal samples. Consistently, the T cell score of the CD group was significantly higher than that of the normal sample (Figure 3B). The above analysis shows that T-cell enrichment in CD samples may contribute to immune dysregulation relative to that in normal samples.

### 2.4. Identification of T Cell-Associated Differentially Expressed Genes in CD

#### 2.4.1. Differentially Expressed Gene Analysis of CD and Normal Samples

In GSE75214 dataset, 3438 differential genes were identified, of which 1856 were upregulated and 1582 were downregulated, respectively (Figure 3C). We performed GO and KEGG pathway enrichment analyses on DEGs and found that they were closely related to Th1 and Th2 cell differentiation, Th17 cell differentiation, PPAR, p53, MAPK, IL-17, FoxO, chemokine, TNF, and TGF-beta signaling pathways (Figure 3D).

#### 2.4.2. WGCNA Analysis between CD and Normal Samples

**Figure 4:**
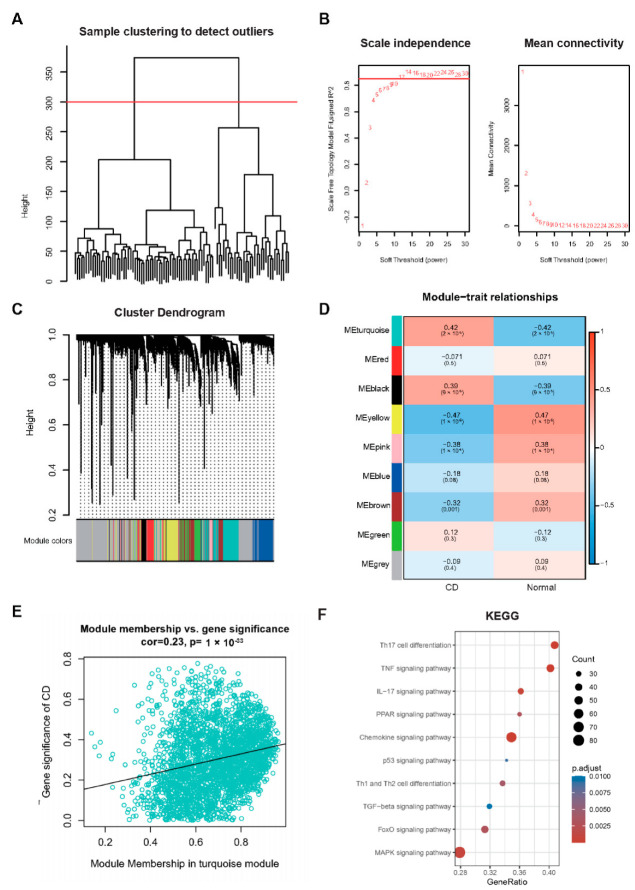
Results of WGCNA analysis. (**A**): Sample clustering analysis. (**B**): Analysis of network topology for various soft-thresholding powers. (**C**): Graph of gene dendrogram and module colors. (**D**): Results of correlation between nine modules and correlation results between sample types. (**E**): Correlation of turquoise modules with CD genes. (**F**): Results of the KEGG annotation part of the turquoise module gene.

We hierarchically clustered GSE75214 dataset and used Pearson correlation coefficient to calculate the distance between genes to construct a weighted co-expression network (Figure 4A). The co-representation network conforms to a scale-free network, implying that, the log(k) of the occurrence of a node with a degree of connection k is negatively correlated with the logarithmic log(P(k)) of the probability of the occurrence of the node, and the correlation coefficient is greater than 0.85. To ensure that the network was scale-free, we chose β to take a value of 12 (Figure 4B). In the next step, we converted the expression matrix into an adjacency and topology matrix and clustered the genes. Following the hybrid dynamic shear tree criterion, we set the minimum number of modules per gene network to 180 and obtained 9 modules (Figure 4C). Notably, grey modules are gene sets that cannot be aggregated. The correlation of each module with CD and normal is shown in Figure 4D. It can be seen that the turquoise and black modules displayed significant positive correlation with CD. For each gene module, we performed KEGG functional enrichment analysis and found that the turquoise module was closely associated with immunity (Figure 4E,F).

### 2.5. Identification of Key Genes and Analysis of Protein Interaction Networks

**Figure 5:**
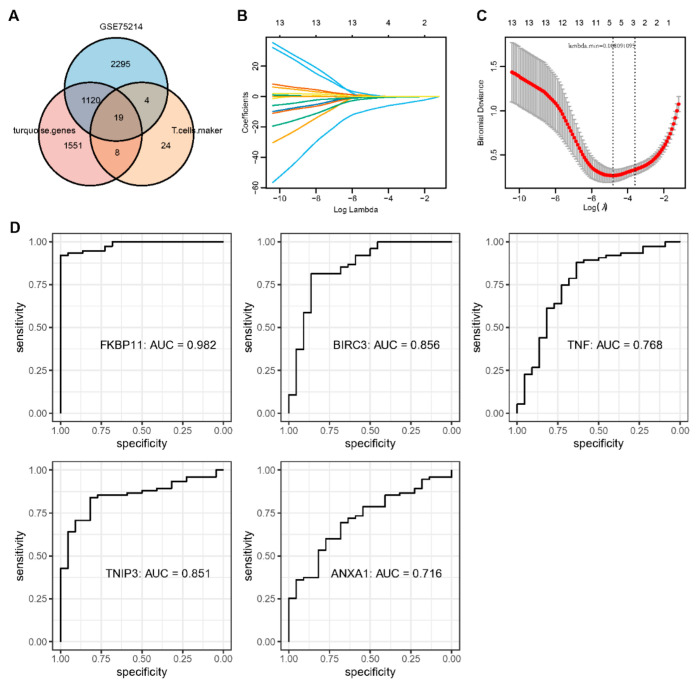
Identification of T cell-specific key genes. (**A**): Intersection of GSE75214, turquoise module and T cell marker genes. (**B**): Changes in independent variables’ trajectories. (**C**): Confidence interval for each lambda. (**D**): Predicted ROC curve of expression values for key genes in GSE75214.

The T cell marker genes, the DEGs identified by GSE75214, and immune module genes were intersected, and we obtained 19 key genes (Figure 5A). Next, we performed LASSO regression to analyze the trajectory of each independent variable (Figure 5B). A gradual increase in lambda corresponded to a gradual increase in the independent variable coefficients tending to zero. The confidence interval of each lambda was analyzed using five-fold cross-validation (Figure 5C). The model reached optimality when lambda = 0.008091095. Consequently, the next step of the analysis focused on the five genes selected for lambda = 0.008091095. ROC curves of all five key genes in the dataset GSE75214 showed excellent predictive efficacy, especially FKBP11 with an AUC value of 0.982 (Figure 5D).

**Figure 6:**
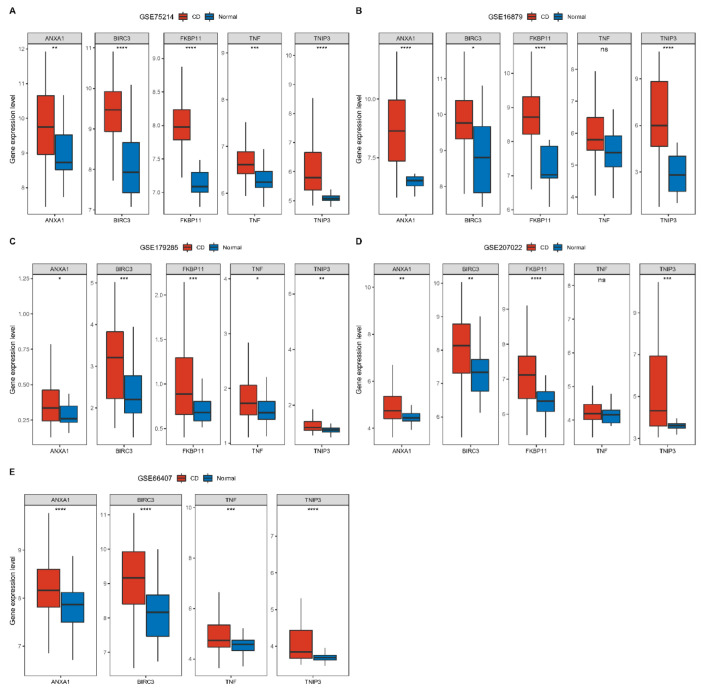
Multiple datasets to validate the expression of key genes, including GSE75214, GSE16879, GSE179285, GSE207022, and GSE66407. (**A**): Expression of ANXA1, BIRC3, FKBP11, TNF and TNIP3 in CD and normal tissues respectively in dataset GSE75214. (**B**): Expression of ANXA1, BIRC3, FKBP11, TNF and TNIP3 in CD and normal tissues respectively in dataset GSE16879. (**C**): Expression of ANXA1, BIRC3, FKBP11, TNF and TNIP3 in CD and normal tissues respectively in dataset GSE179285. (**D**): Expression of ANXA1, BIRC3, FKBP11, TNF and TNIP3 in CD and normal tissues respectively in dataset GSE207022. (**E**): Expression of ANXA1, BIRC3, FKBP11, TNF and TNIP3 in CD and normal tissues respectively in dataset GSE66407. FKBP11 expression was not obtained from dataset GSE66407. (* represents *p* < 0.05, ** represents *p* < 0.01, *** represents *p* < 0.001, **** represents *p* < 0.0001).

In addition, we explored the expression of these five genes in different datasets. Consistently, five genes were expressed higher in CD patients than in normal subjects. Among them, FKBP11 had no expression data in the dataset GSE66407 (Figure 6). We then analyzed network maps of genes interacting with five genes using the GeneMANIA website and performed functional analysis, all showing close association with immunity (Figure S8). ANXA1 is mainly involved in adaptive immune responses based on somatic cell reorganization of immune receptors constructed from immunoglobulin superfamily structural domains. In contrast, there may be genetic interactions between TNIP3 and BIRC3 involved in toll-like receptor-based innate immune responses.

### 2.6. Correlation of Key Genes with Immune Cell Infiltration and Pathways

**Figure 7:**
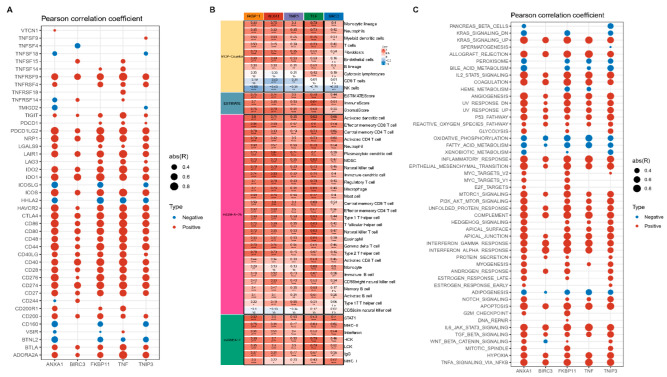
Immune landscape and pathway enrichment of key genes. (**A**) Correlation of key genes with immune genes in dataset GSE75214; (**B**) Correlation of key genes with immune scores in dataset GSE75214; (**C**) Correlation of key genes with pathway scores in dataset GSE75214. (Points shown in the AC plot are significant, non-significant points are blank; * in the B plot represents *p* < 0.05, ** represents *p* < 0.01, *** represents *p* < 0.001, **** represents *p* < 0.0001).

We calculated and visualized Pearson correlations of key genes with immune-related genes in the dataset GSE75214. Key genes demonstrated significant positive correlations with most immune genes, especially immune checkpoint related genes such as CTLA4, CD86, PDCD1LG2, and CD40 (Figure 7A).

Moreover, we evaluated the immune score of the dataset GSE75214 samples using ESTIMATE as well as the MCP-counter algorithm. Additionally, based on the twenty-eight and seven immune cell genes obtained, we scored each sample by the ssGSEA method and calculated the Pearson correlation between key genes and immune scores. There was a significant positive correlation between key genes and immune scores (Figure 7B).

Furthermore, we obtained 50 pathway file h.all.v7.5.symbols.gmt from HALLMARK, and scored and calculated Pearson correlations. Surprisingly, the key genes displayed the same significant positive correlation with the pathway scores (Figure 7C).

### 2.7. Trajectory of T Cell Maturation in CD

**Figure 8:**
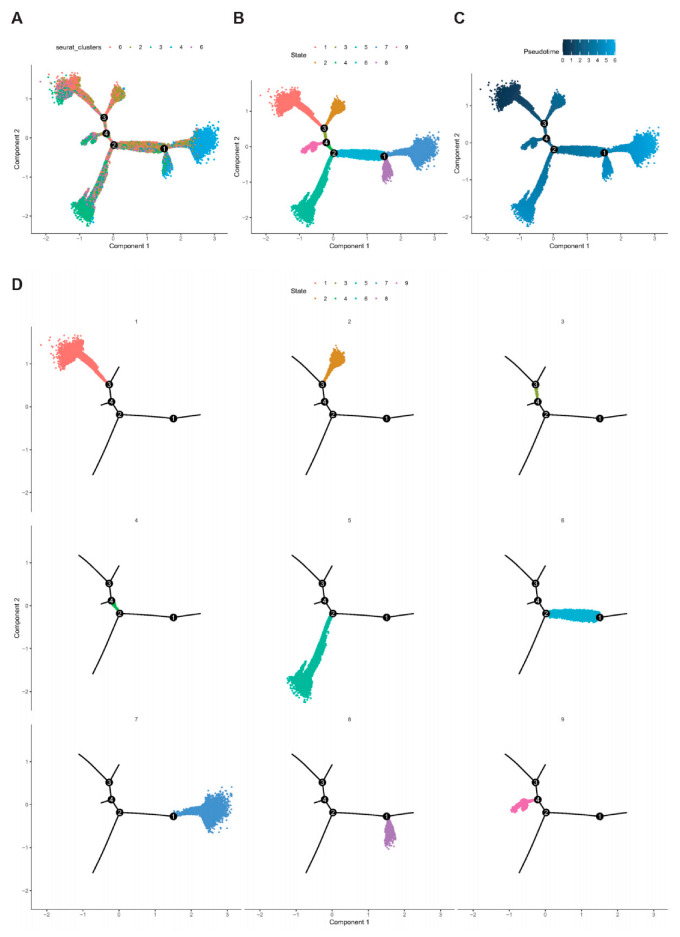
Trajectory analysis of T cells in CD lesions. (**A**): Cell trajectory curve showing different clusters of T cells in CD. (**B**): A plot of the cell trajectory curves shows the distribution of T cells at various stages of CD. (**C**): Display of T-cell developmental timing in CD. (**D**): An illustration of nine subtypes on a trajectory plot. Each dot in the figure represents a cell, and the numbers in the black circles in the figure represent the nodes in the trajectory analysis that determine the different cell states.

Pseudotime analysis using Monocle software (V2.22.0) was performed to further clarify the developmental stages of key genes in T cell subpopulations (. PMID number: 28825705) We found that T cells could roughly differentiate into nine subtypes (Figure 8A,B). A lighter color represents a later stage of development according to the timeline of cell differentiation (Figure 8C). T cells in cluster1 and cluster2 were at an early developmental stage, and cells in cluster7 were at a late developmental stage (Figure 8D). According to their expression at different differentiation periods, we found that ANXA1 expression increased as its development continued to mature. The changes of BRIC3 and FKBP11 were not obvious in the early stage and started to increase in the middle and late stages of development. The degree of changes of TNF and TNIP3 were relatively less obvious.

### 2.8. Construction and Validation of CD Diagnostic Models

**Figure 9:**
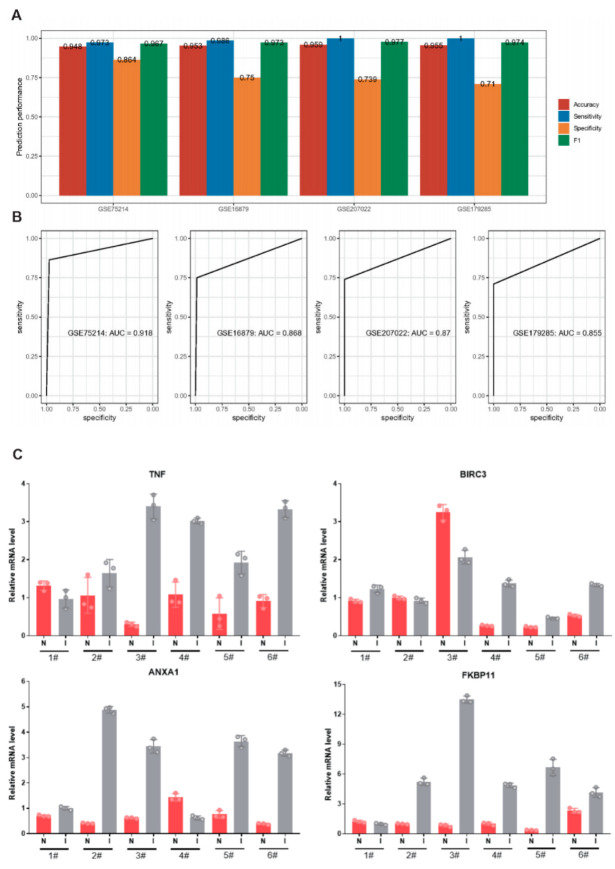
Validation of the diagnostic model on multiple datasets. (**A**): Performance of the diagnostic model on four datasets. (**B**): ROC curves of the diagnostic model on four datasets. (**C**): Expression of TNF, ANXA1, BIRC3, TNIP3, and FKBP11 at mRNA levels in patient tissues. The expression of TNIP3 in patient tissues is shown in the accompanying Figure S9. N: normal; I: Inflammation (CD).

We constructed a diagnostic model for CD based on the five key genes. Meanwhile, we trained the model on the dataset GSE75214 by XGBoost algorithm, followed by validation on three datasets GSE16879, GSE179285, and GSE207022, respectively. The sensitivity was higher than 0.95 and the specificity was higher than 0.71 in all four datasets (Figure 9A). The AUC values of the ROC curves for all four datasets were higher than 0.85 in all four datasets, indicating the reliability of our diagnostic model (Figure 9B). Further, we included six CD patients and extracted diseased tissue and surrounding normal tissue to validate the mRNA levels. Although CD is a heterogeneous autoimmune disease, the expression levels of five key genes (TNF, BIRC3, ANXA1, TNIP3, and FKBP11) were significantly higher in most CD tissues than that in normal tissues (Figure 9C). Therefore, the advantages of our diagnostic model are also reflected from a clinical perspective.

### 2.9. Molecular Docking of Key Genes to Small Molecule Drugs

We used Auto Dock Vina v.1.2.0 to perform molecular simulation docking of target proteins and ligand molecules. The docking algorithm was Lamarck genetic algorithm, the docking method was semi-flexible docking, exhaustiveness was set to eight, and the maximum number of conformations output was set to nine. The docking bonding free energy and the docking results are shown below (Figure S10A). In general, when the binding energy is less than −5 kcal/mol, the binding is excellent, and when it is less than −7 kcal/mol, the binding is strong. We took Azathioprine with BIRC3 and Glucoocorticoid with BIRC3, which had the best binding energy, for visualization and analysis of the docking situation. The docking results of this group were visualized in 3D using PyMOL2.3.0 software, and the products were visualized in 2D using Discovery Studio, and the detailed docking of Azathioprine and Glucoocorticoid with BIRC3 groups is shown in Figure S10B–G.

### 2.10. Gene Susceptibility Analysis to Drugs

**Figure 10:**
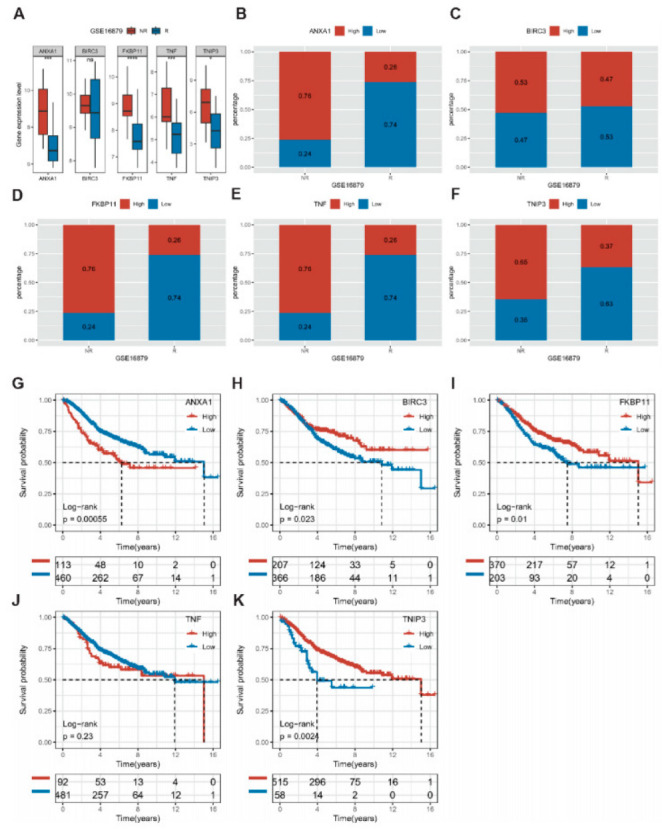
Gene susceptibility analysis of drugs and a prognostic evaluation of IBD-associated colon cancer (GSE39582). (**A**–**F**): Expression of ANXA1, BIRC3, FKBP11, TNF, and TNIP3 in people treated with IFX. (**G**–**K**): The high and low expressions of ANXA1, BIRC3, FKBP11, TNF, and TNIP3 in colon cancer correlates with its prognosis. N: respond to IFX; NR: non-respond to IFX.

We further invoked the 5-gene model to predict gene expression signatures of IFX responses and their drug susceptibility in CD patients (GSE16879). We found that ANXA1, FKBP11, TNF, and TNIP3 were significantly more expressed in the subgroup that did not respond (NR) to IFX than in the subgroup that responded (R) (Figure 10A–F). Even in CD patients who did not respond to IFX, the high expression of ANXA1, FKBP11, TNF, and TNIP3 accounted for more than 65%. Consequently, the expression of the above four genes is increased in the population of CD who do not respond after IFX treatment, further demonstrating that they strongly promote inflammation and may even antagonize the therapeutic effect of IFX.

### 2.11. Association of Key Genes with the Prognosis of Colon Cancer

We analyzed the expression differences of key genes between tumor and normal samples of colon cancer in the GSE39582 as well as TCGA datasets. In the GSE39582 dataset, ANXA1, BIRC3, FKBP11, and TNIP3 were significantly different (Figure S11A). Among them, ANXA1 and FKBP11 were highly expressed in tumor tissues compared to normal tissues, whereas BIRC3 and TNIP3 were lowly expressed in tumor tissues. In the TCGA dataset, only BIRC3 and FKBP11 had significant differences, and the trend was consistent with that in the GSE39582 dataset (Figure S11B). Further, we explored whether these genes were associated with colon cancer prognosis in the datasets GSE39582, GSE17538, and TCGA. We used the surv_cutpoint function of the R package survminer (V0.49) to obtain the optimal cutoff for each gene, and divided the gene expression into high and low expression groups according to cutoff and plotted their KM curves. In the dataset GSE39582, high expression of BIRC3, FKBP11, and TNIP3 was associated with a better prognosis, while high expression of ANXA1 was associated with a worse prognosis (Figure 10G–K). In dataset GSE17538, high expression of TNF and TNIP3 was associated with a better prognosis, while high expression of ANXA1 was associated with a poorer prognosis (Figure S11C). In the dataset TCGA, high expression of TNF and TNIP3 was strongly associated with a better prognosis in colon cancer (Figure S11D). Therefore, in combination with multi-dataset analysis, TNIP3 may be an independent factor in assessing colon cancer prognosis.

## 3. Discussion

As single-cell sequencing technologies continue to iterate, the heterogeneity of IBD has come to the attention of researchers, including different genetic and environmental backgrounds [15]. Currently, about 100 different risk genes and loci are associated with CD or UC, but these polymorphisms account for only 23% and 16% of the heritability of CD and UC, respectively, and the rest may be associated with epigenetic and environmental factors [16,17]. Among these, CD is considered a multifactorial autoimmune disease based on tissue inflammation. Under steady-state conditions, the inflammatory pathways of the intestinal mucosa are often severely restricted. For example, the main symptom of innate immune-mediated susceptibility to celiac disease is intestinal mucus secretion [18]. Moreover, when bacterial antigens induce abnormal activation of adaptive immune cells, the irrepressible immune response forces further aggravation of CD damage [19]. Admittedly, although genetic and environmental differences in CD patients remain elusive, strong evidence for a dysregulated immune response may be attributed to a T cell-mediated inflammatory cascade [20,21,22].

In recent years, several lines of evidence point to the migration of T cells to the intestine, activating and perpetuating intestinal inflammation and tissue destruction [23,24]. Interestingly, the inducers of T-cell intestinal localization are relatively organ-specific, allowing for a higher safety profile of T-cell targeted therapy. In addition, T cells are highly plastic, which may result from a flexible genetic program in response to local environmental cues or cellular heterogeneity [25]. Although some studies have highlighted and developed strategies for T-cell targeting in CD, specific T-cell subsets in CD have not yet been identified. As a result, we lack tools to stratify patients based on pathogenic pathways, leading to the neglect of therapies with high potential value for specific subsets of patients.

In view of this, we identified nine cell types with clear boundaries and their cellular communication based on single-cell data from colonic mucosa of CD patients. Further, we identified T cell-specific key genes and their immune landscape based on the intersection of T cell marker genes, DEGs from CD, normal tissue, and WGCNA results. The TNF-centered 5-gene diagnostic model is not only closely related to KRAS, P53, and IL6/JAK/STAT3 pathways, but also involved in physiological processes such as epithelial mesenchymal transition, coagulation, and apoptosis. Additionally, this 5-gene diagnostic model may also have potential immunotherapeutic synergistic effects, with positive correlations with immune checkpoints such as CTLA4, CD86, PDCD1LG2, and CD40. We completed a 5-gene-based diagnostic model for the molecular docking of small molecule drugs, which may herald a new diagnostic pathway for CD and an initial exploration of specific high-potential therapies. Based on the magnitude of molecular docking binding energy, we selected five genes, TNF, ANXA1, BIRC3, TNIP3, and FKBP11, for validation in CD patient tissues. The QPCR results corroborate the high heterogeneity of CD, with each gene having a distinct and differential expression in different patients. However, despite the heterogeneity, the expression of key genes was higher in most CD tissues than in normal tissues. This also echoes the superiority of our diagnostic model.

Needless to say, although anti-TNF antibodies have become the first choice of biological therapy for many CD patients, such as ADA and IFX, some patients still have difficulty responding to or tolerating them [26,27,28]. Moreover, the remitting efficacy of anti-TNF antibodies diminishes over time, often leading to a loss of secondary response. In response to this clinical question, a retrospective cohort study by Christopher et al. found that more than 50% of CD patients treated with IFX and ADA experienced secondary loss of response [29]. Interestingly, another open clinical trial proposed that ADA was well-tolerated and effective in maintaining clinical remission in CD patients who failed IFX therapy [30]. Therefore, the necessity of intervening with a second anti-TNF agent after failure of the first anti-TNF therapy is of great clinical interest to explore. A retrospective study from France included 61 CD patients who were re-exposed to IFX after receiving and discontinuing IFX and ADA [31]. The remission rate at weeks 6–8 after IFX reintroduction was 42%, which predicted a better long-term response (*p* = 0.006). Indeed, the likelihood of retention of treatment was 60% and 51% at 12 and 24 months after IFX reintroduction (median treatment duration of 16 months), respectively. Another study included 118 patients with CD who received anti-TNF therapy for the first time (median duration of treatment was 5 months), of whom 64 patients showed no response and 54 patients had only a partial response [32]. After these patients received a second anti-TNF treatment, 44% of the first-time non-responders achieved remission and 59% of the first-time partial responders achieved clinical remission. Both studies strongly confirm the heterogeneity of anti-TNF drugs in CD patients and that the initial drug failure response does not completely negate the secondary drug failure response.

However, a meta-analysis noted that ADA had a significantly higher clinical remission rate than ustekinumab and vedolizumab, and a higher safety profile [33]. Therefore, the efficacy and safety of different biologic agents after failure of the first anti-TNF treatment deserve to be considered. Surprisingly, ADA was effective in promoting mucosal healing as well as fistula closure, both in patients with CD without biologic therapy and those who failed anti-TNF therapy [34,35].

Consequently, searching for new drug targets to optimize the efficacy of anti-TNF is a challenging proposition. Molecular docking has become a valuable tool for exploring protein structures. Molecular docking can be used before and after the docking of a protein with a specific ligand to more accurately evaluate the magnitude of the affinity between the ligand and the receptor by calculating the free energy of binding. We performed molecular docking of five genes, TNF, ANXA1, BIRC3, FKBP11, and TNIP3, with azathioprine, glucocorticoids, and mesalazine to verify the binding properties of the target proteins and the three ligands. We found that BIRC3 and ANXA1 bind extremely well to glucocorticoids. Although glucocorticoids are effective in relieving symptoms of CD in the real world and are used at high rates, international guidelines for the management of IBD recommend reducing their use given the risk of complications. In addition, we found that the binding energy of BIRC3 and azathioprine was second only to that of binding to glucocorticoids. A study published in Gut found that azathioprine monotherapy was less effective in patients with CD than in those with UC (*p* < 0.0001), even after a multivariate analysis was performed (OR 0.47,95%CI 0.43–0.51, *p* < 0.0001) [36]. However, Peter Laszlo Lakatos et al. reported that early intensive application of the immunosuppressant azathioprine appeared to modify the natural course of CD and reduce the risk of surgical resection.

A prospective randomized controlled study enrolling 90 patients with IBD who had failed ADA/IFX treatment (CD = 48) found that combining a TNF inhibitor and azathioprine had higher rates of clinical failure-free survival and pharmacokinetic failure-free survival than TNF inhibitor alone [37]. This is an important encouragement for patients with secondary failure to respond due to immunogenicity after first anti-TNF therapy, suggesting that a second anti-TNF agent in combination with azathioprine is a potential option.

It is worth mentioning that inflammation is similar to a “double-edged sword” and that the exact molecular mechanism of the “inflammatory-cancerous” transformation remains unclear [38]. On the one hand, the majority of inflammatory cells in acute inflammation prevent pathogenic and tumor invasion and promote tissue repair. In addition, inflammatory factors can also activate the immune system and enhance the body’s cancer-suppressive effects [39]. On the other hand, chronic inflammation produces cytokines that cause abnormalities in inflammatory pathway transmission by inducing genetic mutations [40]. In addition, chronic inflammation provides the basis for tumor progression through the recruitment of various immunosuppressive cells (MDSC, Treg, etc.) to establish an immunosuppressive tumor microenvironment [41,42]. For example, our group previously found that small heat shock protein CRYAB significantly inhibited the secretion of inflammatory factors by macrophages, thereby suppressing intestinal inflammation [43]. However, CRYAB was reported to promote the progression of colon cancer [44]. The discrepancy between the above two studies suggests that the same molecule may exercise different biological functions in inflammation and tumor, which may be related to immunosuppression. If suppression is excessive, it may increase the risk of carcinogenesis. Therefore, the functional differences between the molecule in the figure in inflammation and tumor may be attributed to its inhibition of immune cells, thereby increasing the likelihood of malignancy.

Unfortunately, there are still some limitations to this study. Firstly, the model was constructed based on a public dataset and large-scale prospective clinical validation is inevitable and desirable. Secondly, this study did not involve in vitro or in vivo experiments, and the potential molecular mechanisms of T cells in CD are unclear. Thirdly, other immune cell subtypes, including B cells, natural killer cells, fibroblasts, and mast cells may play a surprising role in CD. However, these cells have not been addressed in our study. Therefore, the road to molecular drug generation is still a long way for us.

In the future, in-depth exploration of the pathophysiological mechanisms of CD from a cell subpopulation perspective is expected to become mainstream for precision and targeted therapies. Targeting strategies based on specific T-cell subsets in CD will provide clinicians with the confidence to identify potential biomarkers and accurately stratify patients. New drug targets will continue to optimize the efficacy of existing biologics and compensate for the heterogeneous deficiencies of CD therapy, such as loss of secondary response and intolerance. This certainly brings encouragement to CD patients, especially those who do not respond to anti-TNF therapy.

## 4. Materials and Methods

### 4.1. Data Sources and Preprocessing

#### 4.1.1. Single-Cell Data Sources and Processing

The single-cell dataset for CD was derived from GSE134809 in GEO database (https://www.ncbi.nlm.nih.gov/geo/ (accessed on 16 January 2023)), with a total of 22 samples [45]. We used R package Seurat (V4.0.5) to read and filter single-cell data, and used Percentage_Feature_Set function to calculate the mitochondrial proportion [46]. Based on this, we ensured that each cell expressed more than 250 genes and less than 20% mitochondrial content. Each cell has a UMI greater than 800 and log10GenesPerUMI greater than 0.8. The 22 samples before numerical quality control had a total of 112,245 cells, and 72,793 cells remained after quality control (Figures S1 and S2, Table S1).

#### 4.1.2. Crohn’s Disease Expression Profile Data Sources and Processing

The expression profiles and clinical information of CD and control samples were derived from five datasets, including GSE75214, GSE16879, GSE179285, GSE66407, and GSE207022 [47,48,49]. For different datasets, we convert probes to gene symbols based on annotation files built into the datasets and retain CD and control samples.

#### 4.1.3. Colon Cancer Expression Profile Data Sources and Processing

RNA-seq data for colon cancer and clinical follow-up data were derived from the TCGA database (https://portal.gdc.cancer.gov/ (accessed on 16 January 2023)). For RNA-seq data, we convert FPKM to TPM and perform log2 processing. We downloaded raw data from the GEO database for two datasets, GSE17538 and GSE39582 [50,51], which hold sequencing data from the GPL570 platform. We use the RMA function of R package affy (V1.66.0) for the processing and standardization of expression spectrum data [52]. Finally, we retained colon cancer tumor samples with survival time, survival status, and normal samples.

### 4.2. Single-Cell Clustering and Cell Annotation Analysis

We performed single-cell analysis using R package Seurat (V4.0.5) and performed cell identification by annotating maker genes.

### 4.3. Cell Communication Analysis

Using CellChatDB.human (V 1.6.0) as a reference, we communicated cells through CellChat to analyze cell-to-cell interactions and crosstalk of 32 pathways between cells [53].

### 4.4. Differential Gene Identification and Analysis

According to the threshold |FC| > 1.2 and FDR < 0.05, we performed R package limma to identify the differentially expressed genes (DEGs) in CD and normal samples and performed GO and KEGG pathway enrichment analysis.

### 4.5. WGCNA and Functional Enrichment Analysis

We performed a co-expression analysis using WGCNA for GSE data and KEGG functional enrichment analysis for the genes in each module using R package ClusterProfiler (v4.2.2) [54,55]. Detailed parameters and results are analyzed below.

### 4.6. Correlation of Key Genes with Immunity and Pathways

The ESTIMATE algorithm was obtained from the website (https://sourceforge.net/projects/estimateproject/ (accessed on 20 January 2023)) to estimate stromal and immune fractions based on biomarkers associated with stromal cell and immune cell infiltration in the sample [56]. Immunity and matrix scores predict the levels of stromal and immune cells (ImmuneScore and StromalScore) and calculate the relevance of key genes to them. The MCP-counter method for the robust quantification of the absolute abundance of eight immune cells and two stromal cell populations in heterogeneous tissues from transcriptome data (T cells, CD8 T cells, Cytotoxic lymphocytes, B lineage, NK cells, monocytic lineage, myeloid dendritic cells, and neutrophils) calculates the correlation of key genes with them [57]. Gene set variation analysis (GSVA) is a nonparametric, unsupervised gene set enrichment method that estimates scores for certain pathways or markers based on transcriptome data [58]. We used ssGSEA method in GSVA to evaluate the genes of 28 acquired immune cells and seven immune-related pathway genes, and calculated the correlation of key genes with them [59]. In addition, we calculated and visualized the Pearson correlation of key genes with immune-associated genes in the dataset GSE75214 [60,61,62,63].

### 4.7. Establishment and Validation of Diagnostic Models

We used the XGBoost algorithm to train the model on dataset GSE75214 and validated it using GSE16879, GSE179285, and GSE207022 [64,65]. Since the dataset GSE66407 lacked a gene, we did not adopt it. On the training set, we used 10× cross-validation, max_depth = 2, subsample = 0.7, colsample_bytree = 0.4, num_class = 2, objective = ‘multi:softprob’, nrounds = 1000.

### 4.8. Real-Time Quantitative Polymerase Chain Reaction (RT-qPCR)

Six tissue specimens from CD patients (both normal and diseased tissues) were collected from Xinhua Hospital, Shanghai Jiaotong University School of Medicine. The Ethics Committee of Shanghai Xinhua Hospital approved the study and the patients provided informed consent in writing. Total RNA was extracted from tissue samples of six CD patients using TRIzol reagent according to the manufacturer’s instructions. cDNA was synthesized using PrimeScript™ RT Reagent Kit and SYBR Green qPCR Mix (Vazyme Biotech, Nanjing, China). The CFX Connect Real-Time System (Bio-Rad, Hercules, CA, USA) was used to complete amplification in 40 cycles. Relative mRNA expression was evaluated using the 2^−ΔΔCt^ method and normalized to the expression of *GAPDH*. The sequences of the PCR primers used in this study are as follows: ANXA1 (forward: 5′-GCGGTGAGCCCCTATCCTA-3′, reverse: 5′-TGATGGTTGCTTCATCCACAC-3′), BIRC3 (forward: 5′-AAGCTACCTCTCAGCCTACTTT-3′, reverse: 5′-CCACTGTTTTCTGTACCCGGA-3′), FKBP11 (forward: 5′-AGACACGCTTCACATACACTACA-3′, reverse: 5′-TCGCTTCTCTCCCACACAC-3′), TNIP3 (forward: 5′-CTCCTCATCCAAAACGGTTCACC-3′, reverse: 5′-TGCTCCGTAGAACTTTCTGCGG-3′), TNF (forward: 5′-CTCTTCTGCCTGCTGCACTTTG-3′, reverse: 5′-ATGGGCTACAGGCTTGTCACTC-3′), and GAPDH (forward: 5′-GTCTCCTCTGACTTCAACAGCG-3′, reverse: 5′-ACCACCCTGTTGTTGCTGTAGCCAA-3′).

### 4.9. Molecular Docking

PDB data (https://www1.rcsb.org/ (accessed on 5 February 2023)) and the Alpha-Fold database were used to identify and download target proteins for key genes. We downloaded the molecular structure files for Azathioprine (PubChem CID:2265), Glucocorticoid (PubChem CID:46937290), and mesalazine (PubChem CID:4075) from Pubchem database. We deleted water molecules and proligands from the downloaded target protein with PyMOL2.3.0, and used Chem3D (2020 version) to perform molecular mechanical optimization of the optimal conformation of the ligand molecule, and finally obtained the optimal conformation with minimal energy. We hydrotreated the pretreated target protein molecules with Auto Dock Tools 1.5.6 to hydrogenate the ligand molecules of the optimal conformation obtained by molecular mechanics optimization and determined the twistable bonds. We used the POCASA protein activity pocket online prediction tool to predict the protein activity pocket, set the docking range in the predicted active pocket, and saved the docking range information for formal docking.

### 4.10. Association of Key Genes with the Prognosis of Colon Cancer

We analyzed differences in the expression of key genes between colon cancer and normal samples. Simultaneously, in order to evaluate whether the five genes are related to prognosis in colon cancer, we used R package survminer (V0.49) to obtain the best cutoff of each gene and divided it into high or low expression groups, plotting its survival curve.

## 5. Conclusions

In recent years, an increasing number of studies have explored potential prognostic markers of disease using single-cell sequencing data, facilitating accurate patient stratification and identifying patients sensitive to treatment. In this study, we focused on targeting T cell-related signature molecules in CD patients based on single-cell sequencing to construct a diagnostic model, in which abnormal immune infiltration is also an important feature of CD activation. Further, TNF, ANXA1, BIRC3, FKBP11, and TNIP3 as central genes can be used to predict potential drug targets, providing a unique perspective for CD diagnosis and drug development.

## Data Availability

The datasets used during the current study are available from the corresponding author upon reasonable request.

## Supplementary Materials

The following supporting information can be downloaded at: https://www.mdpi.com/article/10.3390/ijms24076054/s1.
