# Supplementary material for: Single-Cell Sequencing-Based Validation of T Cell-Associated Diagnostic Model Genes and Drug Response in Crohn’s Disease
Source: Int J Mol Sci. 2023 Mar 23;24(7):6054. doi: 10.3390/ijms24076054 (PMC10093907; doi:10.3390/ijms24076054)
Supplement: Supplementary file 1 [file ijms-24-06054-s001.zip › Supplementary materials.pdf]

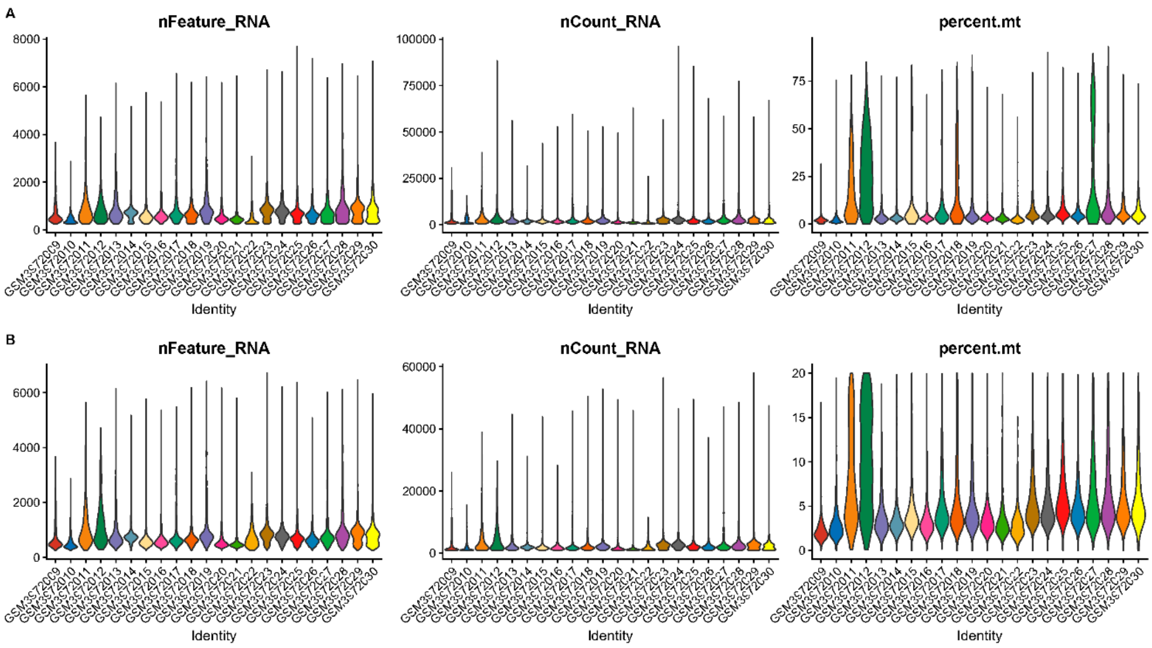

Figure S1: A: Visualization of cellular features before quality control of single cell datasets. B: Visualization of cellular features after quality control of single cell datasets.

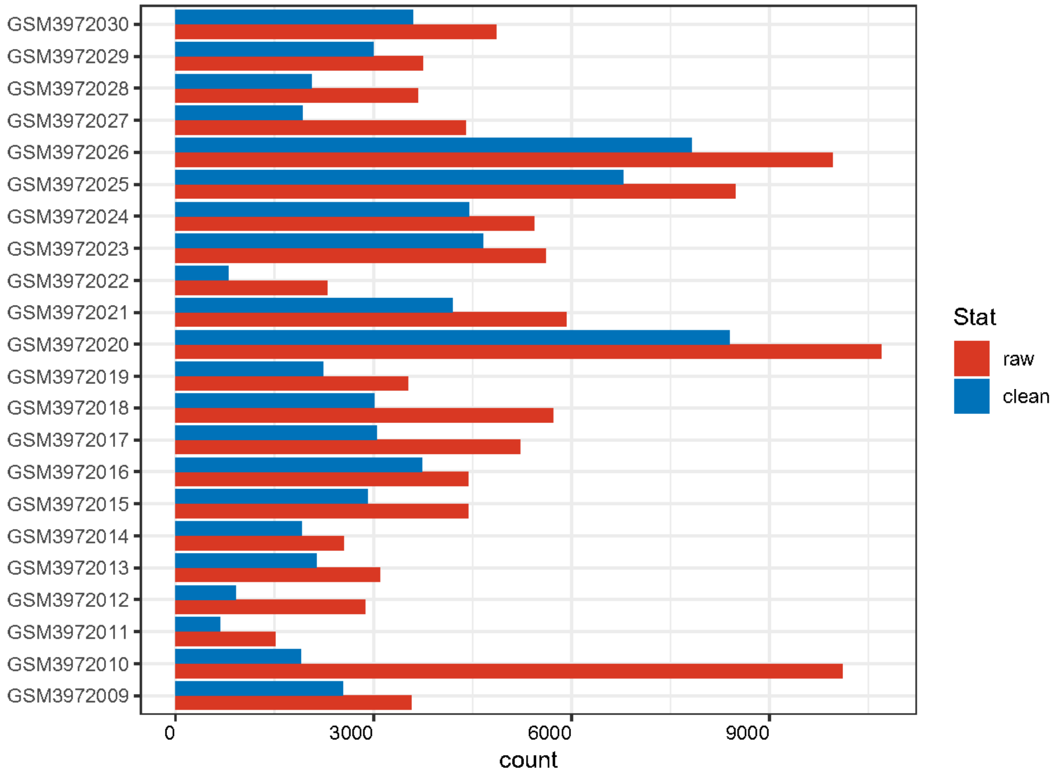

Figure S2: Comparison of cell numbers before and after data cleaning for single-cell data sets.

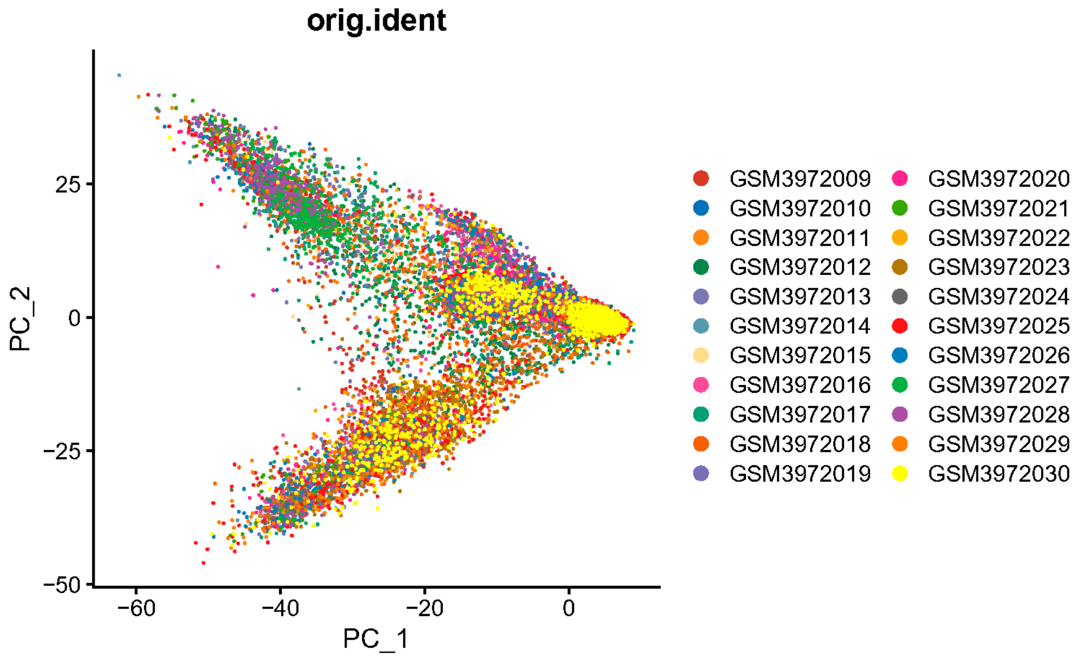

Figure S3: Eliminate batch effects between samples.

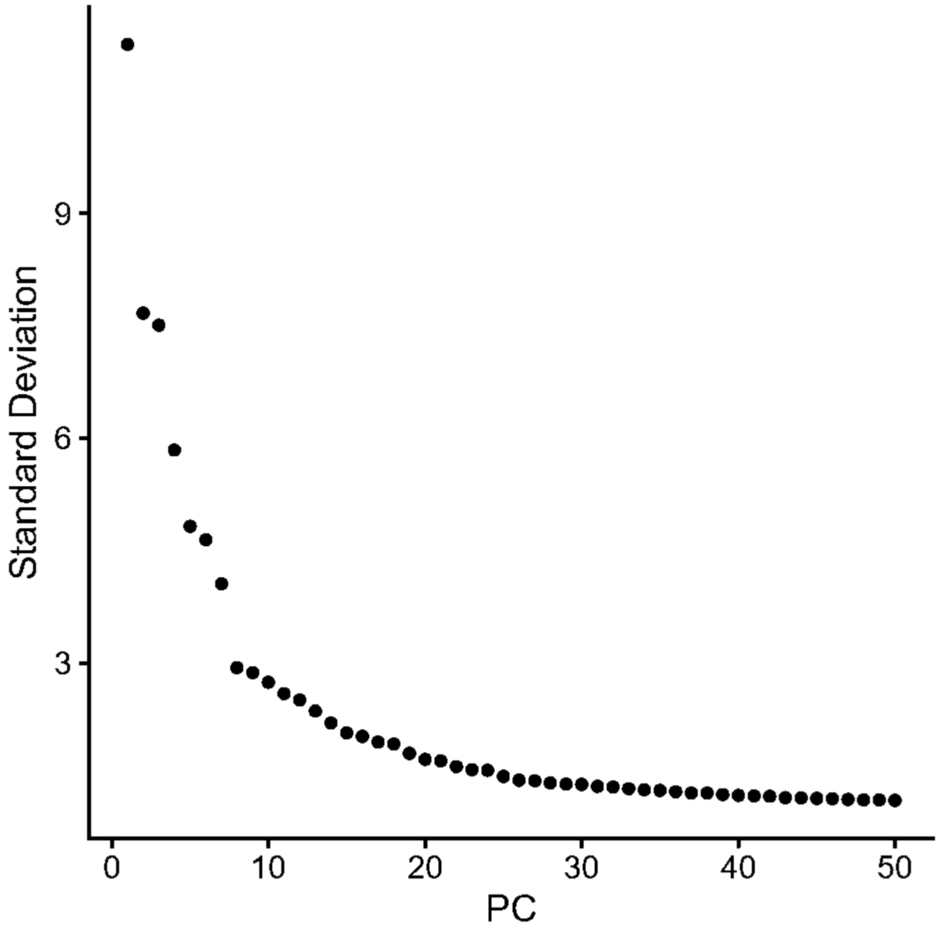

Figure S4: Perform PCA downscaling to find anchor points.

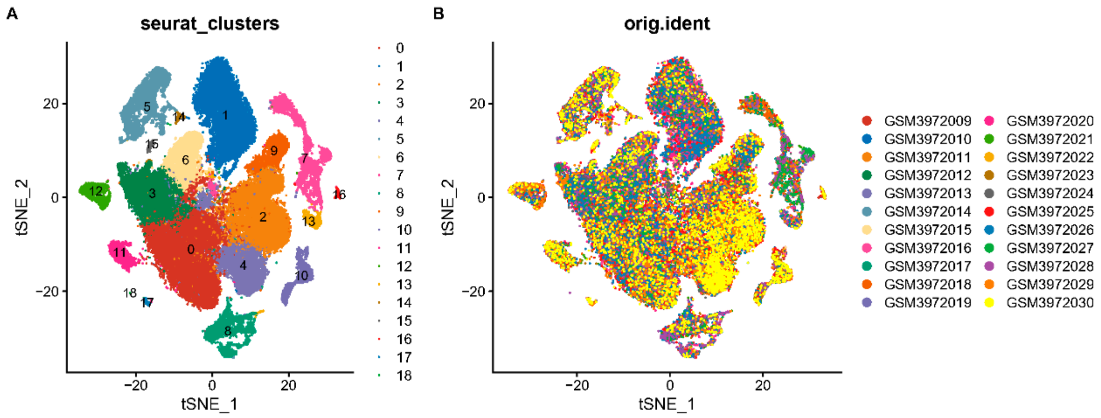

Figure S5: Nineteen subgroups were obtained by cell clustering analysis.

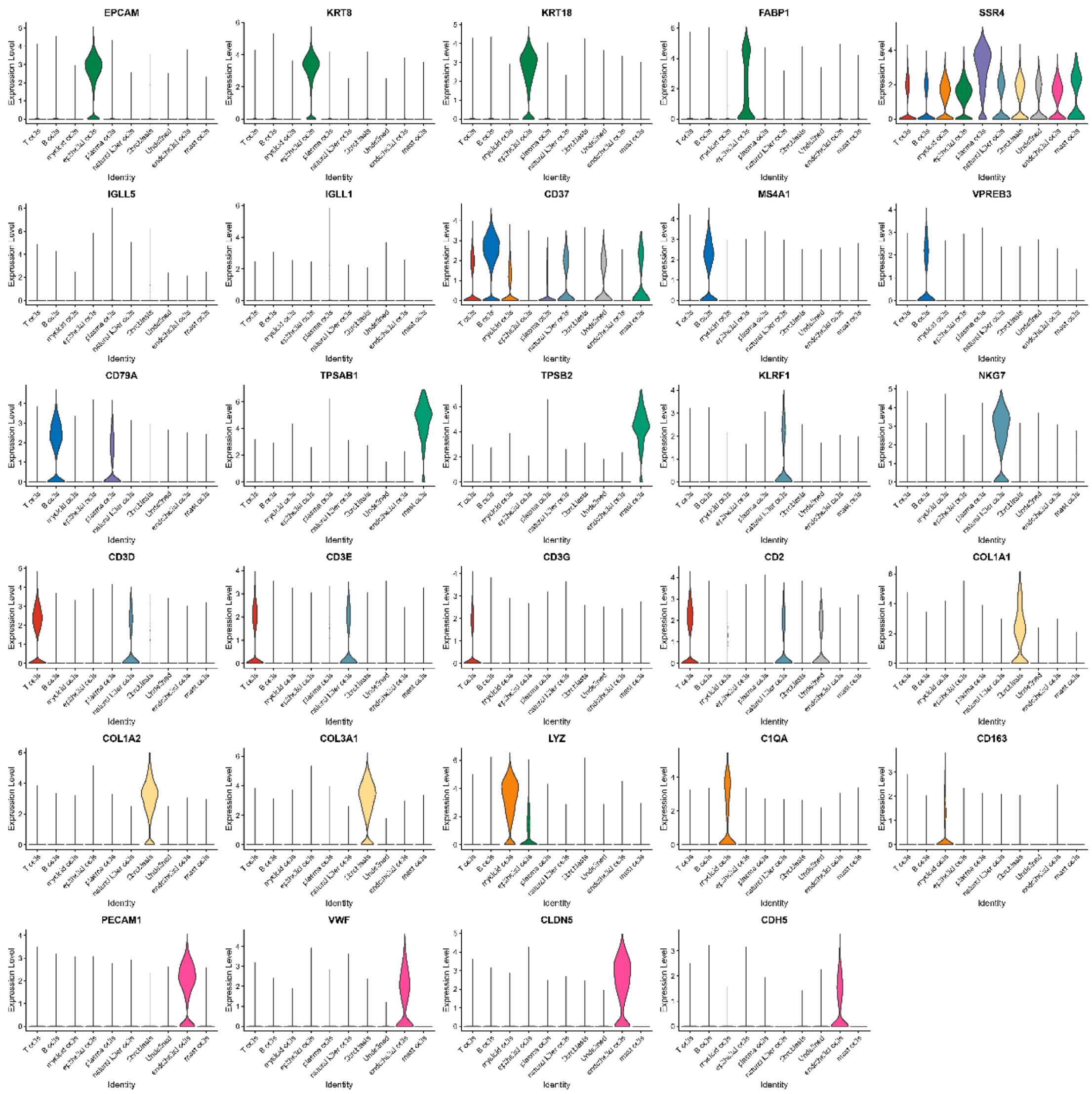

Figure S6: Expression of marker genes in various cells.

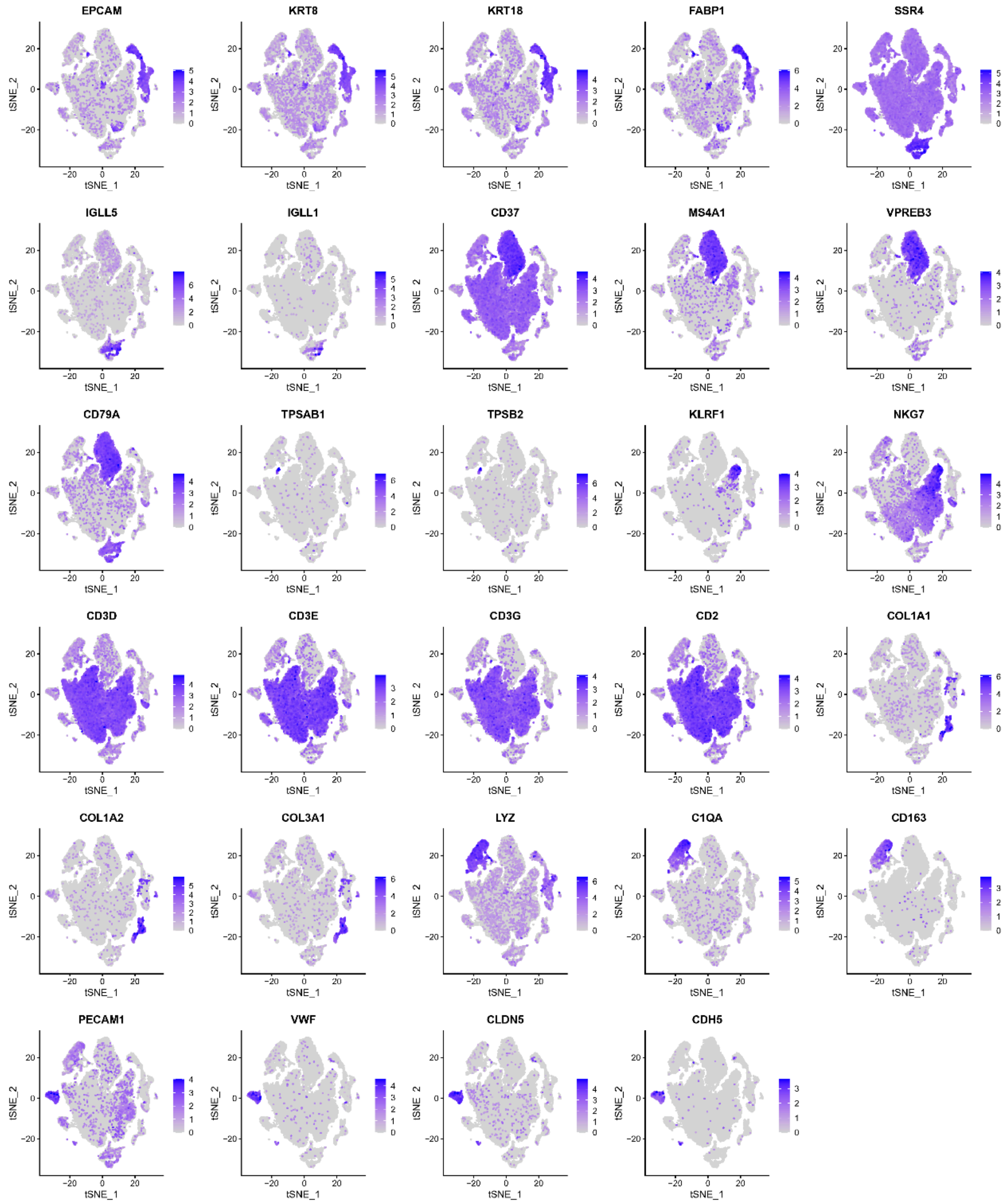

Figure S7: Distribution of post-descending expression of marker genes in various cells.

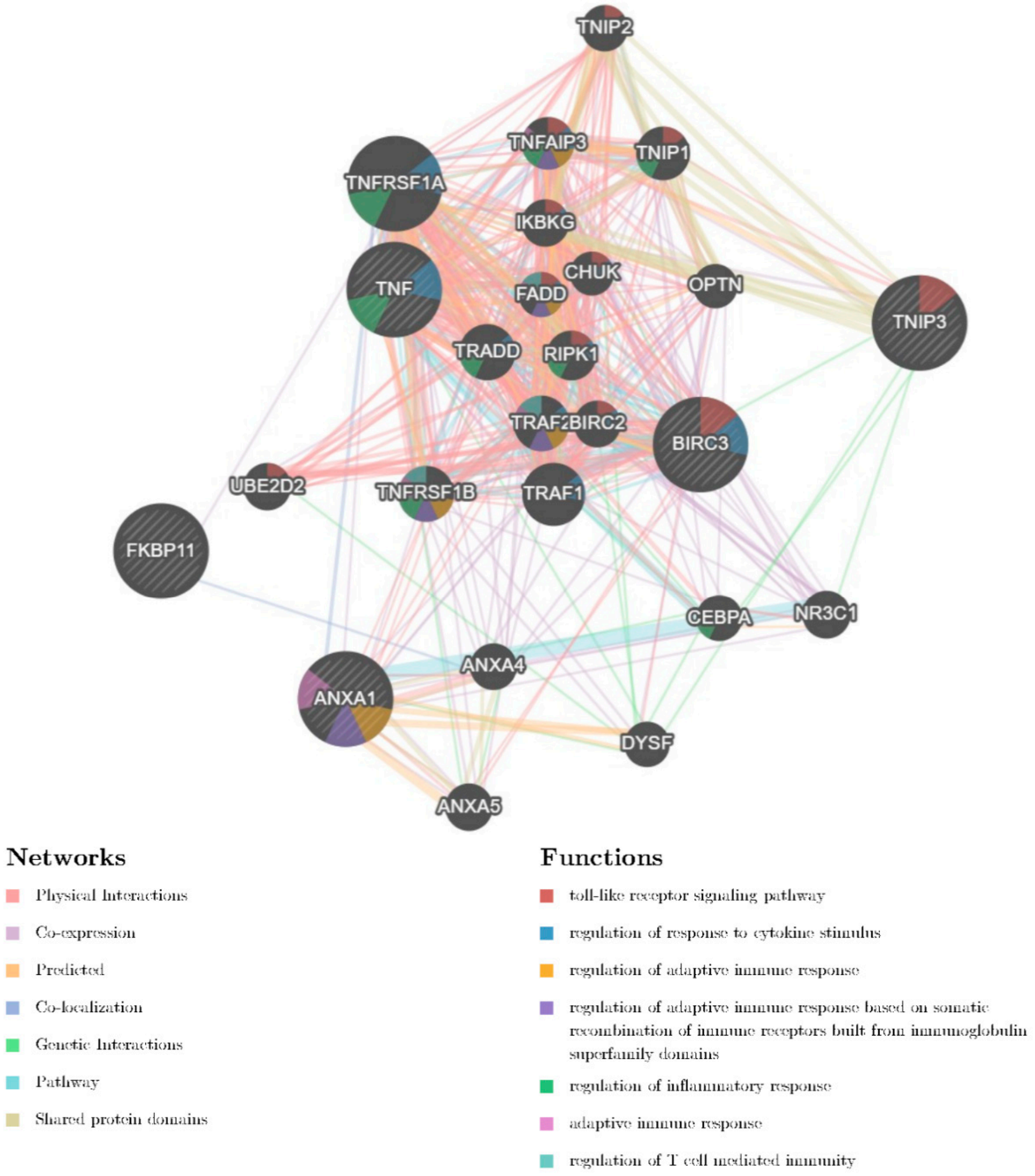

Figure S8: Schematic representation of protein interactions of TNF, ANXA1, BIRC3, FKBP11 and TNIP3.

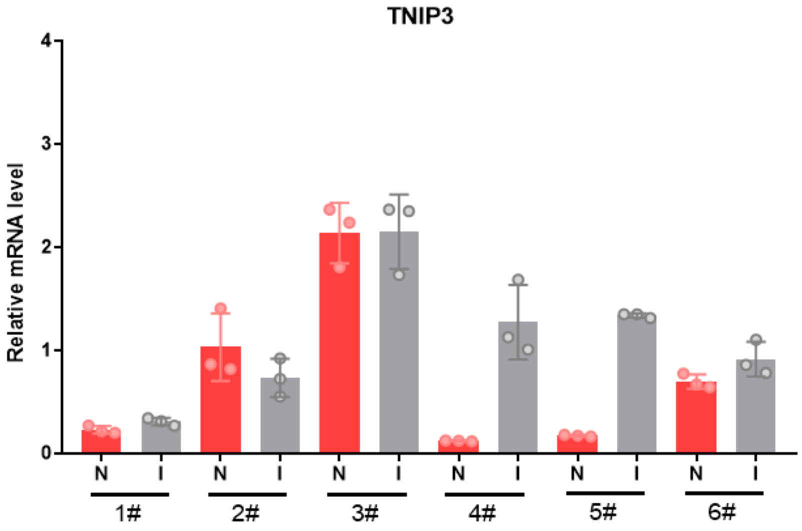

Figure S9: Expression of TNIP3 at mRNA levels in patient tissues.

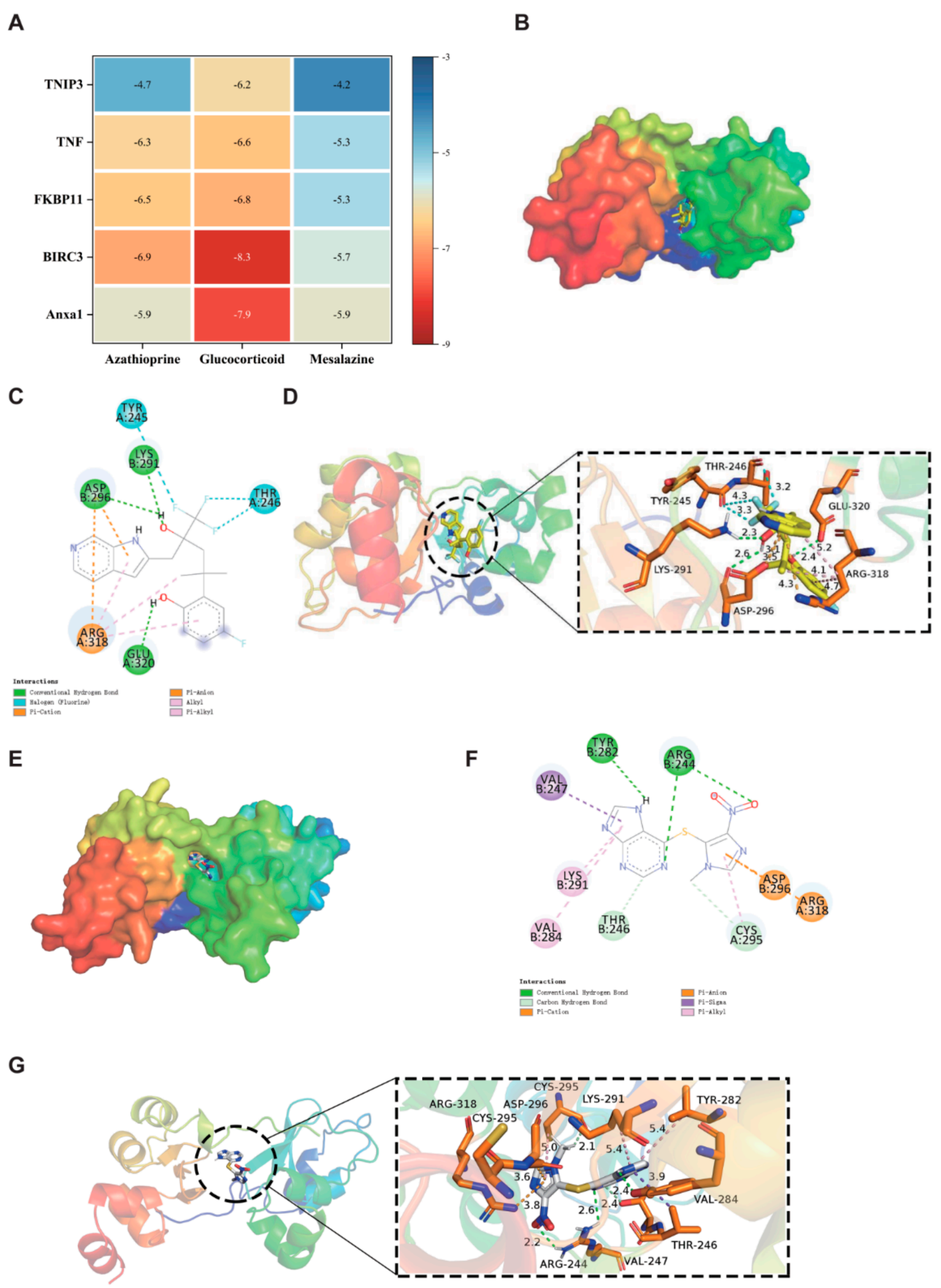

Figure S10: Binding efficacy of key genes to small molecule drugs. A: Binding energy data for key genes with Azathioprine, Glucoocorticoid and Mesalazine. B-D: Analysis of the binding force of Glucoocorticoid to the target protein BIRC3, including 3D, 2D (the different forces and binding modes generated by docking are indicated by different colored dashed lines and explained) and a local zoomed-in view of the interaction of the drug with the amino acids in the target. E-G: Analysis of the binding force of Azathioprine to the target protein BIRC3, including 3D, 2D (the different forces and binding modes produced by docking are indicated by different colored dashed lines and explained) and a local zoomed-in view of the interaction of the drug with the amino acids in the target.

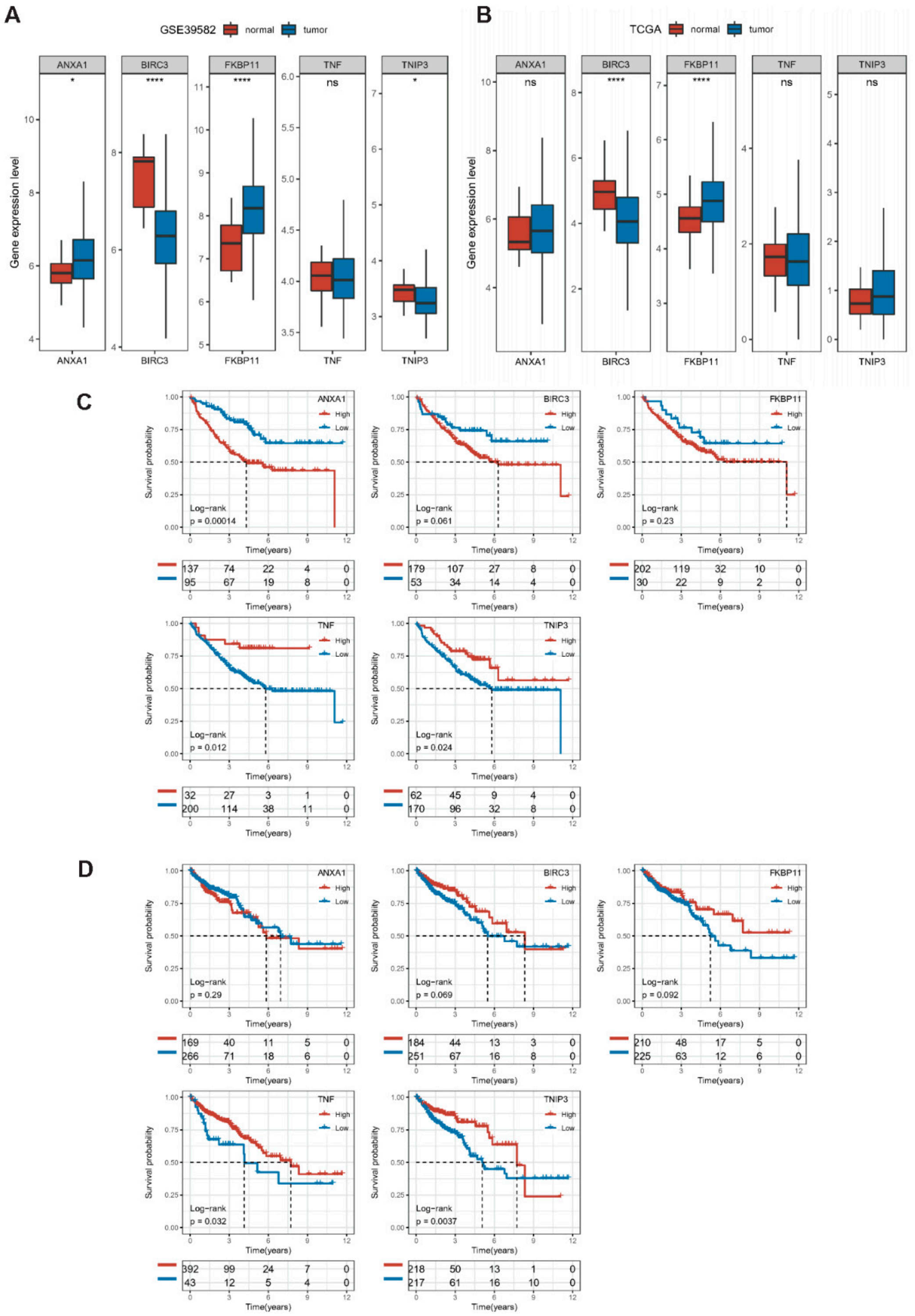

Figure S11: A: Differential expression of ANXA1, BIRC3, FKBP11, TNF and TNIP3 in tumor tissues and normal tissues in the dataset GSE39582. B: Differential expression of ANXA1, BIRC3, FKBP11, TNF and TNIP3 in tumor tissues and normal tissues in the dataset TCGA.C-D: Prognostic evaluation of five T cell-specific key genes in IBD-associated colon cancer (GSE17538 and TCGA).
